# NK cell-derived exosomes enhance the anti-tumor effects against ovarian cancer by delivering cisplatin and reactivating NK cell functions

**DOI:** 10.3389/fimmu.2022.1087689

**Published:** 2023-01-19

**Authors:** Heyong Luo, Yanhua Zhou, Jing Zhang, Yingchun Zhang, Shiqi Long, Xiaojin Lin, Anqing Yang, Jiangyao Duan, Na Yang, Zhiru Yang, Qiyuan Che, Yuxin Yang, Ting Guo, Dan Zi, Weiwei Ouyang, Wei Yang, Zhu Zeng, Xing Zhao

**Affiliations:** ^1^ Tissue Engineering and Stem Cell Experiment Center, Guizhou Medical University (GMU), Guiyang, Guizhou, China; ^2^ Department of Immunology, College of Basic Medical Sciences, Guizhou Medical University, Guiyang, Guizhou, China; ^3^ Department of Biology, School of Basic Medical Sciences, Guizhou Medical University, Guiyang, Guizhou, China; ^4^ Department of Life Sciences, Faculty of Natural Sciences, Imperial College London, London, United Kingdom; ^5^ Department of gynecology, The Affiliated Hospital of Guizhou Medical University, Guiyang, China; ^6^ Department of gynaecology and obstetrics, Guizhou Provincial People's Hospital, Guiyang, China; ^7^ Department of Thoracic Oncology, The Affiliated Hospital/The Affiliated Cancer Hospital of Guizhou Medical University, Guiyang, China; ^8^ Department of Oncology, Guizhou Medical University, Guiyang, China; ^9^ Key Laboratory of Infectious Immune and Antibody Engineering of Guizhou Province/Engineering Research Center of Cellular Immunotherapy of Guizhou Province/Department of Biology and Engineering, College of Basic Medical Sciences, Guizhou Medical University, Guiyang, China

**Keywords:** ovarian cancer, exosomes, natural killer cells, cisplatin, immunomodulatory

## Abstract

Exosomes are membranous vesicles actively secreted by almost all cells and they deliver certain intracellular molecules, including nucleic acids, proteins, and lipids, to target cells. They are also considered to be good carriers for drug delivery due to their biocompatibility, high permeability, low immunogenicity, and low toxicity. Exosomes from immune cells were also reported to have immunomodulatory activities. Herein we evaluated the application of exosomes derived from expanded natural killer cells (eNK-EXO) for the treatment of ovarian cancer (OC). We demonstrate that eNK-EXO express typical protein markers of natural killer (NK) cells, can be preferentially uptaken by SKOV3 cells, and display cytotoxicity against OC cells. Furthermore, eNK-EXO loaded with cisplatin could sensitize drug-resistant OC cells to the anti-proliferation effect of cisplatin. In addition, we show that eNK-EXO could activate NK cells from immunosuppressive tumor microenvironment, the mechanism of which is explored by transcriptional analysis. In summary, eNK-EXO exhibit anti-tumor activity against OC on its own, could be used to deliver cisplatin and enhance its cytotoxic effect against drug-resistant OC cells and also reverse the immunosuppression of NK cells, which may lead to great prospect of using eNK-EXO in the treatment of OC in the clinic. Our work also builds a strong foundation for further evaluation of eNK-EXO in other solid tumor therapies.

## Introduction

Ovarian cancer (OC) has the highest mortality rate among gynecological malignancies, and most patients are not diagnosed until late stages due to insidious or nonspecific early clinical symptoms (1). Surgery combined with platinum-based chemotherapy has been the primary treatment for OC. Although most patients are sensitive to platinum-based first-line chemotherapy, more than 80% of patients relapse within 18 months of initial treatment and become resistant to almost all chemotherapy drugs (2). Therefore, primary or secondary resistance has become the main contributing factor to the high mortality rate of OC. Unfortunately, the mechanisms for drug resistance in OC remain unclear. In recent years, increasing evidence has shown that the occurrence of acquired drug resistance of tumor cells is closely related to tumor microenvironment (TME) (3–6). In OC, the tumor is mainly confined to the abdominal cavity, where the peritoneal fluid provides mobile and accessible dynamic environment between OC cells and stromal cells, making OC more prone to recurrence, metastasis, and drug resistance (7). Therefore, the development of novel and efficient therapeutic strategies resolving the drug resistance of OC is expected to improve the prognosis of OC patients (8, 9).

Exosomes are nano-scale bilayer vesicles actively secreted by various cells and contain diverse biomolecules (10). Recently, it has been discovered that exosomes mediate near and long-distance cell-to-cell communication in both healthy and diseased cells, affecting all aspects of cell biology (11, 12). Different cells exert cell-to-cell communication by releasing exosomes carrying different components, such as nucleic acids, lipids, proteins, and metabolites (13). These exosomes are taken up by the recipient cells and information is delivered through material exchange or release of contents (13). In recent years, the potential application of exosomes in anti-cancer therapy have attracted increasing attentions (12). Exosomes derived from NK cells (NK-Exo) encapsulate perforin, granzyme, microRNA (e.g., miR-186, miR-3607, etc.), and other tumor-killing substances during biogenesis, thus exhibiting cytotoxic effects on a variety of tumor cells, including breast cancer, melanoma, and neuroblastoma (14–18). Moreover, NK-Exo express typical NK cell markers (e.g. CD56) and cytotoxicity receptors (e.g.,NKG2D) (10, 19), and can deliver the chemotherapy drug paclitaxel for breast cancer therapy (20). A previous study showed that exposure to an immunosuppressive environment mimicked by TGF-β and IL-10 did not attenuate the original affinity and anti-tumor activity of NK-Exo (10, 16). It could be that NK-Exo retain their anti-tumor activity because of lacking the signalling and metabolic pathways that respond to inhibitory TME (21). Therefore, impeded by many inherent limitations of NK cell-based therapies, such as insufficient tumor targeting, limited NK cell infiltration in TME, and inhibition of NK cell function by TME (22), scientist found that NK-Exo may provide an alternative for cancer treatment, especially in solid tumors, as a “cell-free” immunotherapeutic strategy.

Based on the advantages of drug delivery and immunomodulatory activity of exosomes, we hypothesized that exosomes derived from expanded NK cells (eNK) can deliver tumor therapeutic drugs and reverse the immunosuppression of NK cells. In this study, we demonstrate the therapeutic effect of exosomes derived from eNK cells (eNK-EXO) against OC *in vitro* by itself and with loaded cisplatin (DDP). eNK-EXO not only sensitize OC cells to the cytotoxic effect of DDP, but also reactivate NK cells after being suppressed by TME. These findings suggest that eNK-EXO could potentially reverse immune suppression by reactivating defective NK cells in TME, enhancing the therapeutic effect of anti-cancer drugs.

## Materials and methods

### Human samples

The cord blood mononuclear cells (CBMC) were isolated from the cord blood of 3 healthy donors by density gradient centrifugation. eNK were obtained by co-culturing CBMC with irradiation-inactivated K562 engineered cells for 14 days in the presence of IL-2 (23). The human ascites mononuclear cells were isolated from the ascites of 2 patients with malignant OC by Ficoll gradient density centrifugation, and the NK cells in ascites (AS_NK) were obtained using EasySep™ Human NK Cell Isolation Kit (Stemcell Technologies). AS_NK and eNK cells were maintained in RPMI 1640 medium supplemented with 5% human plasma and 1% penicillin/streptomycin (Gibco, Gaithersburg, MD, USA). All cells were cultured at 37°C in a humidified atmosphere of 5% CO_2_-95% air. The studies involving human samples were reviewed and approved by the Ethical Committee of GuiZhou Medical University, Guizhou, China (2022–82). The patients/participants provided their written informed consent to participate in this study.

### Cell culture and treatment

The human OC cell line SKOV3, OV-90 and COC1/DDP (cisplatin resistant) were obtained from the China Center for Type Culture Collection. NK92/MI cells and human ovarian epithelial cells IOSE80 were obtained from Procell Life Science&Technology Co., Ltd. SKOV3, OV-90, COC1/DDP and IOSE80 were cultured in Mc-Coy’s 5a medium, DMEM/F12 medium (Gibco, Gaithersburg, MD, USA), RPMI 1640 medium (Gibco), and DMEM/High Glucose medium (HyClone, USA), respectively. All cultural media were supplemented with 10% fetal bovine serum (FBS) and 1% penicillin/streptomycin (Gibco). NK92/MI was cultured in MEM-Alpha medium (Gibco) supplemented with 12.5% FBS, 12.5% horse serum, 0.2mM Inositol, 2-Mercaptoethanol and 0.02 mM folic acid (Sigma-Aldrich, St. Louis, MO, USA). All cells were cultured at 37°C in a humidified atmosphere of 5% CO_2_-95% air.

To obtain NK cells treated with OC ascites (AS-t-NK), the ascites collected from 7 patients (**Supplementary Table S1**) with malignant OC were aliquoted into 50-mL conical tubes and centrifuged at 300 g for 10 min to separate cell pellets from supernatant (24). eNK cells were then cultured in RPMI 1640 medium containing 10% OC ascites for 24 h.

### Preparation of eNK-EXO and eNK-EXO-DDP

eNK-EXO were isolated using differential ultracentrifugation method (10). eNK cells were cultured in conditioned medium containing 5% exosome-free FBS for 48 h and the supernatants were centrifuged for 10 min at 300 g and 10 min at 2000* g* to remove cells and large debris. Next, the supernatant was centrifuged at 10000 g for 30 min and filtered through a 0.22 μm filter (Millipore) to remove large vesicles before further centrifugation at 100000 g for 70 min with P28S rotor (Hitachi, CPN100NX, Japan). The pellets were then resuspended in PBS and centrifuged again at 100000 g for 70 min. The exosome pellet was resuspended in 100 μL PBS. All of the above steps were performed at 4°C. The obtained exosomes were quantified by BCA protein assay (Beyotime, China) and stored at -80°C to use within two months.

To prepare DDP loaded eNK-EXO (eNK-EXO-DDP), we used electroporation to load DDP (Qilu Pharmaceutical, China) into eNK-EXO (25). We first mixed 250 µg of eNK-EXO, 250 µg of DDP and 100 µL electroporation buffer in a 200 μL electroporation cuvette, and then the mixed sample was electroporated at 450 V for 120 ms (Celetrix) (26, 27). The obtained eNK-EXO-DDP was used immediately.

### Transmission electron microscopy

To examine exosomes under transmission electron microscopy, the purified exosomes (>10^9^/mL) from the supernatant of NK cells were suspended in PBS and mounted onto the copper grid. Excess liquid was gently removed with filter paper. Uranyl acetate was then loaded onto the copper grid for 1 min and excess liquid was gently removed with filter paper. Samples were air dried and observed with the HT-7700 transmission electron microscope (Hitachi, Japan) at 100 kV.

### Nano-flow cytometry analysis

The particle concentration, size distribution and phenotypes of eNK-EXO were analyzed by nFCM (NanoFCM, China) according to reported protocols (28, 29). Briefly, two single photon counting avalanche photodiodes were used for the simultaneous detection of the side scatter and fluorescence of individual particles. The instrument was calibrated for particle concentration using 200 nm PE and AF488 fluorophore-conjugated polystyrene beads and for size distribution using Silica Nanosphere Cocktail (NanoFCM Inc., S16M-Exo). Any particles that pass by the detector within a 1-min interval were recorded in each test. All samples were diluted to attain a particle count within the optimal range of 2000~12,000/min. Using the calibration curve, the flow rate and side scattering intensity were converted to the corresponding vesicle concentration and size on the NanoFCM software (NanoFCM Profession V1.0).

For immunofluorescent staining, the following antibodies were purchased from Biolegend: APC-conjugated mouse anti-human CD16 (clone 3G8), FITC-conjugated mouse anti-human CD107a (clone H4A3), PE-conjugated mouse anti-human CD56 (clone MEM-188) and PE-conjugated mouse anti-human CD69 (clone FN50). An aliquot of eNK-EXO samples was suspended in 20 μL of PBS with a particle concentration of 1×10^9^ particles/mL, mixed with 20 μL antibody and incubated for 60 min at 37°C. After incubation, the mixture was washed with PBS and centrifuged at 100,000 *g* for 70 min at 4°C. The pellet was then resuspended in 50 μL of PBS for phenotype analysis.

### Exosome uptake

For the uptake assays, eNK-EXO were labeled with PKH67 (Sigma-Aldrich, USA) and centrifuged at 100,000 g for 70 min to remove any free dye. SKOV3 cells were co-cultured with 20 μg of PKH67-labeled eNK-EXO for 6 h. Then the cells were washed in PBS and fixed in 4% paraformaldehyde, the cell nuclei were stained with 10 μg/mL DAPI. For blocking experiments, SKOV3 cells were co-cultured with 20 μg of PKH67-labeled eNK-EXO for 6 hours in the presence of anti-human CD63 (clone EPR5702; 1:1000; Abcam) or anti-human CD81 (clone D3N2D; 1:1000; Cell Signaling Technology). The cells were then treated with the same steps as described above. For selective uptake detection, SKOV3 cells were labeled by 10 μM DiR (Perkin Elmer, USA) and co-cultured with IOSE80 cells at a ratio of 1:1. The cells were then incubated with 20 μg of PKH67-labeled eNK-EXO for 6 h at 37°C and treated with the same steps as described above. All the images above were acquired by fluorescence microscope (Olympus BX51) and the fluorescence intensity of intracellular eNK-EXO was analyzed using Image J 1.53a software (National Institutes of Health, USA).

### Cell viability assays

To evaluate the cytotoxicity of eNK-EXO against tumor cells, SKOV3, COC1/DDP and ISOE80 cells were seeded at 1×10^4^ cells per well in 96-well plates and co-cultured with different concentrations of eNK-EXO (10, 20, 40, 60, 80, 100 µg/mL) for 24 h at 37°C. In order to evaluate whether exosomes derived from other cells are cytotoxic against OC cells, we tested the cytotoxicity of 10 µg/mL exosomes derived from mesenchymal stem cells (MSC-exo) on SKOV3 and OV-90 ovarian cancer cells and IOSE80 ovarian epithelial cells as a control. To evaluate the cytotoxicity of eNK-EXO-DDP against tumor cells, SKOV3, COC1/DDP and OV-90 cells were seeded at 1×10^4^ cells per well in 96-well plates. Equal amounts of eNK-EXO, DDP and eNK-EXO-DDP (10µg/mL) were added to tumor cells and co-cultured at 37°C for 24 h. 10 μL of detection reagents from Cell Counting Kit-8 (CCK-8; Dojindo, Japan) were added to cultured cells and incubated at 37°C for 2 h before optical densities (ODs) were measured at 450 nm. Measurements were performed in triplicate for each experiment, and all experiments were repeated three times. Cell viability was calculated by the following formula:


$$\text{Survival rates\% }=\;({\text{OD}}_{\text{experiment}}-{\text{OD}}_{\text{blank}})/({\text{OD}}_{\text{control}}-{\text{OD}}_{\text{blank}})\times 100\%$$

To evaluate the cytotoxicity of NK cells under various treatment, NK cells, NK92/MI cells and AS-t-NK cells were first treated with or without 80 µg/mL eNK-EXO for 24 h. SKOV3 cells were seeded at 1×10^4^ cells per well in 96-well plates, then NK cells, NK92/MI cells, and AS-t-NK cells with or without eNK-EXO treatment were added at an effector-target ratio(E:T) of 1:1 and 2:1, respectively. After 5 h incubation, 10 μL CCK-8 were added into each well for 2 h and the ODs were measured at 450 nm. Measurements were performed in triplicate for each experiment, and all experiments were repeated three times. Killing rate was calculated by the following formula:


$$\text{Killing rates\% }=\left[1-({\text{OD}}_{\text{experiment}}-{\text{OD}}_{\text{effect}})/{\text{OD}}_{\text{control}}\right]\times 100\%$$

### EdU cell proliferation assay

Cell proliferation was determined by EdU Cell Proliferation Kit (RiboBio, China). SKOV3 cells were incubated with 50 μM EdU for 5 h, then fixed with 4% formaldehyde and stained according to the manufacturer’s instructions. The images were acquired by fluorescence microscope (Olympus BX51). The number of proliferating cells and total cells was determined by red and blue signals, respectively. The proliferation rates were calculated by dividing the numbers of proliferating cells by the numbers of total cells.

### Flow cytometry

SKOV3, COC1/DDP cells were pre-treated with 10 µg/mL of eNK-EXO, DDP or eNK-EXO-DDP for 24 h. Cell apoptosis was evaluated by flow cytometry analysis using Annexin V-FITC/PI apoptosis detection kit (Absin, China). Briefly, cells were washed in PBS twice and suspended in 1× binding buffer, incubated in the dark with FITC-Annexin-V for 10 min, followed by PI for 5 min before flow cytometry analysis. Cell cycle assay was carried out using cell cycle detection kit (KeyGEN Biotech, China). Briefly, cells were fixed by 70% ethanol overnight, washed in 1×PBS and treated with RNase-containing propidium iodide for 30 min before flow cytometry analysis. To measure cell proliferation, SKOV3 cells were labeled with 2.5 μM CFSE dye solution for 20 min in the dark and then cultured with the same conditions as above for 48 h after removing free dye. Stained cells were collected on a FC500 flow cytometer (Beckman Coulter, USA) and the data was analyzed by the FlowJo-10 software.

### Western blot analysis

eNK-EXO treated cells were lysed in 1×RIPA buffer with protease inhibitor PMSF. The lysate was mixed with loading buffer and boiled for 10 min. Proteins were separated on a 12% SDS-PAGE gel, transferred to PVDF membranes and blocked by 5% BSA for 2 h. The membranes were incubated with primary antibodies overnight at 4°C. After being washed in 1×TBST, the membranes were incubated with HRP-conjugated goat anti-rabbit IgG (1:5000; Absin) secondary antibodies at room temperature for 2 h. Primary antibodies used above include anti-CD63 (clone EPR5702; 1:1000; Abcam), anti-CD81 (clone D3N2D; 1:1000; CST), anti-TSG101 (clone EPR7130(B); 1:1000; Abcam), anti-Calnexin (1:1000; Biodragon), anti-CD56 (clone E7X9M; 1:1000; CST), anti-perforin (1:1000; Biodragon), anti-Granzyme B (1:1000; Biodragon), anti-cleaved caspase 3 (clone 5A1E; 1:1000; CST), anti-cleaved caspase 7 (clone D6H1; 1:1000; CST), anti-PARP (1:1000; CST) and anti-cleaved PARP (clone D64E10; 1:1000; CST). Anti-β-actin (1:500; Santa Cruz) was used to normalize relative expression of target proteins. The images were visualized by chemiluminescence (Bio-Rad Laboratories, USA).

### Enzyme-linked immunosorbent assay

To measure the production of perforin, TNF-α, CXCL9, CXCL10 and CXCL11, the supernatant was collected and centrifuged to remove remaining cells. The levels of perforin and the above cytokines were evaluated by human perforin ELISA Kit (Enzyme-linked Biotechnology Co. Ltd., Shanghai, China), human TNF-α ELISA Kit (Multisciences, Hangzhou, China), Human CXCL9 ELISA Kit (Multisciences, Hangzhou, China), Human CXCL10 ELISA Kit (Multisciences, Hangzhou, China), and Human CXCL11 ELISA Kit (Multisciences, Hangzhou, China) according to the manufacturers’ instructions, respectively. The ODs were measured at 450 nm and the concentrations of perforin and the cytokines were determined according to their corresponding standard curves.

### RNA-seq

Total RNAs were extracted using TRIzol reagent (Invitrogen, CA, USA) and reverse transcribed into cDNA. The libraries were constructed using VAHTS Universal V6 RNA-seq Library Prep Kit. The transcriptome sequencing and analysis were conducted by OE Biotech Co., Ltd. (Shanghai, China). The libraries were sequenced on an illumina Novaseq 6000 platform and 150 bp paired-end reads were generated. Fragments Per Kilobase of exon model per Million mapped reads (FPKM) of each gene was calculated and the read counts of each gene were obtained by HTSeq-count. Principal component analysis (PCA) analysis were performed using R (v 3.2.0) to evaluate the biological duplication of samples. Differential expression analysis was performed using the DESeq2, q value< 0.05 and foldchange > 2 or foldchange< 0.5 was set as the threshold for significantly differential expression gene (DEGs). Hierarchical cluster analysis of DEGs was performed using R (v 3.2.0) to demonstrate the expression pattern of genes in different groups and samples. Based on the hypergeometric distribution, KEGG pathway enrichment analysis of DEGs were performed to screen the significant enriched term using R (v 3.2.0).

### Statistical analyses

All experiments were repeated at least three times. All statistical analyses were performed using GraphPad Prism 8, and the data are expressed as the mean± SEM. A P value< 0.05 was considered statistically significant by using the Student’s *t*-test: *p< 0.05, **p< 0.01 and ***p< 0.001.

## Results

### Isolation and characterization of exosomes from *ex vivo* NK cell culture

**Figure 1:**
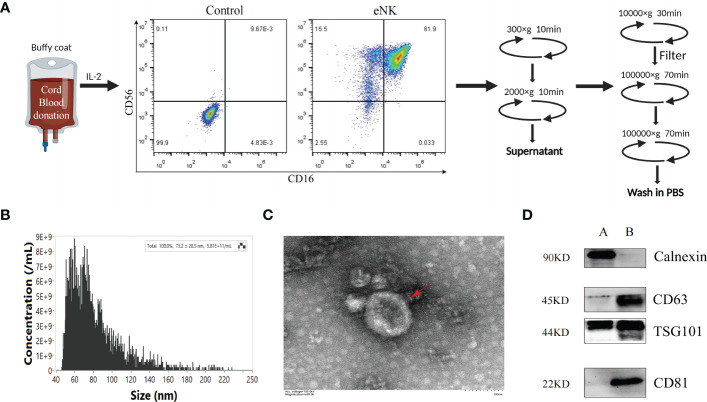
Preparation and Characterization of eNK-EXO. **(A)** Work flow of eNK-EXO isolation. **(B)** Particle size distribution curve of eNK-EXO by NanoFCM. **(C)** Transmission electron microscopy images of eNK-EXO, Bar =100 nm. **(D)** Western blot analysis of CD81 (22 kDa), CD63 (45 kDa), TSG101 (44 kDa) and Calnexin (90 kDa) expression on eNK-EXO. NK cell lysate was used as control. (A: cell lysate, B: eNK-EXO).

We previously established a method to obtain eNK cells from PBMC by co-culturing with K562-mBIL-21 feeder layer cells (Lifeark, China) for 2 weeks (23). Compared with other allogeneic cell sources, such as bone marrow and peripheral blood, cord blood (CB) is readily available as a frozen, “off the shelf” product, which provides more advantages as the starting material for cell therapies. CB is a rich source of immature NK cell population and the NK cells expanded from CB can mature into potent NK cells with higher proliferation rate and cytotoxicity than that expanded from peripheral blood (30–33). It can be reliably produced for clinical use (34). Thus, we can obtain a large number of highly cytotoxic exosomes from eNK cells culture supernatants. In this study, we used mononuclear cells derived from CB to obtain a large number of eNK cells. As shown in **Figure 1A**, on day 14, the culture contained over 95% eNK cells (CD3^-^, CD56^+^, CD16^+^). In order to prepare eNK-EXO, we continued to culture eNK cells in RPMI-1640 with 5% exosome-free FBS. eNK-EXO were then isolated as described in methods. Particle size and concentration analysis by nFCM revealed that the particles were homogeneous in size with an average of 73.2 ± 28.5 nm in diameter (**Figure 1B**). Morphology imaging of eNK-EXO by transmission electron microscopy showed a typical “saucer shape” with a particle size of about 80 nm, which was consistent with nFCM results (**Figure 1C**). Further characterization of eNK-EXO by western blot analysis indicated the existence of exosome markers such as CD81, CD63, and TSG101, while the negative marker calnexin was only detected in cell lysates (**Figure 1D**). Therefore, according to the size, concentration, morphology, and protein markers, we concluded that the eNK-EXO isolated from eNK cell culture by our protocol were of good quality and can be used for following studies.

### eNK-EXO are cytotoxic against OC cell lines

**Figure 2:**
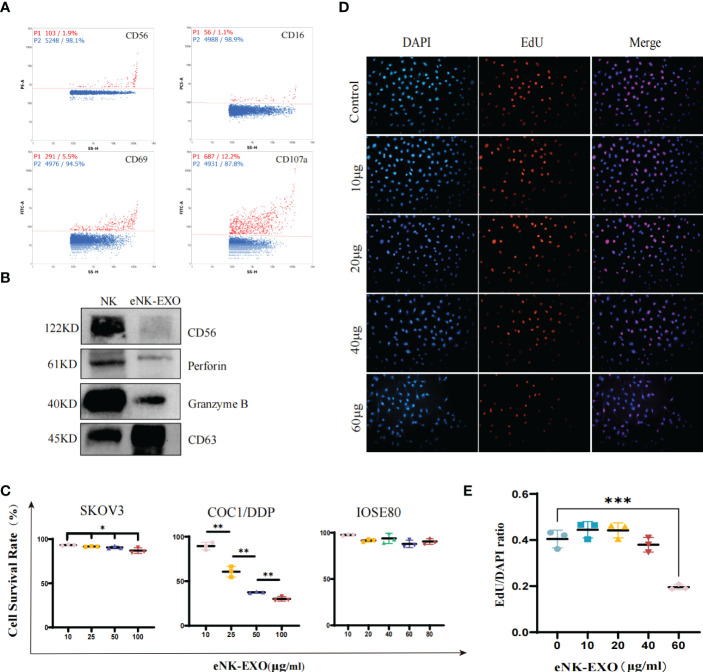
Functional characterization of eNK-EXO *in vitro*. **(A)** Expression of eNK-EXO surface markers CD56, CD16, CD69 and CD107a by NanoFCM. Red dots represent positive signals. **(B)** Western blot analysis of CD56 (122KD), perforin (61KD) and GranzymeB (40KD) expression. NK cell lysate was used as control. **(C)** CCK-8 assay results of eNK-EXO against SKOV3, COC1/DDP and IOSE80 cells (n=3, mean ± SEM, t-test, *p< 0.05, **p< 0.01). **(D, E)** EdU assay results of eNK-EXO against SKOV3 (20×, scale bar = 100 µm). Red signals represent newly proliferating cells, whose proportion was quantified and shown (n=3, mean ± SEM, ***p< 0.001) **(E)**.

To further characterize the isolated eNK-EXO, we first determined whether they contained typical markers and cytotoxic proteins derived from eNK cells. As shown in **Figure 2A**, nFCM analysis showed the presence of CD69 (a marker for NK cell activation) and CD107a (a marker for NK cell degranulation) in eNK-EXO, as well as CD16 and CD56, markers of NK cells. Western blot analysis also confirmed the presence of these two cytotoxic proteins, perforin and granzyme B, in the isolated eNK-EXO (**Figure 2B**). These results were consistent with previous reports on exosomes isolated from NK cell culture medium (19). NK cells are known to exert their cytolytic effect through the release of effector molecules such as perforin and granzymes, which may contribute to the cytotoxicity of eNK-EXO against tumor cell lines.

NK-Exo were reported to cause lysis of breast cancer and melanoma cell lines (15, 18). However, as far as we know, whether eNK-EXO have cytotoxic effect on OC cells has not been studied. Therefore, we evaluated the cytotoxicity of eNK-EXO against OC cell lines by two separate assays. Firstly, the results of the CCK-8 cell viability assay showed that eNK-EXO caused lysis of SKOV3 and COC1/DDP OC cells in a dose-dependent manner, but not IOSE80, the human ovarian epithelial cells (**Figure 2C**). Meanwhile, MSC-exo showed no cytotoxicity on SKOV3, OV-90 and IOSE80 cells (**Supplementary Figure S1**). Secondly, EdU proliferation assay showed that eNK-EXO inhibit the proliferation of SKOV3 cells in a dose-dependent manner (**Figures 2D, E**). The results of two assays cross-validated the cytotoxicity of eNK-EXO against OC cell lines and were safe against healthy ovarian epithelial cells.

### eNK-EXO are preferentially uptaken by OC cell lines

**Figure 3:**
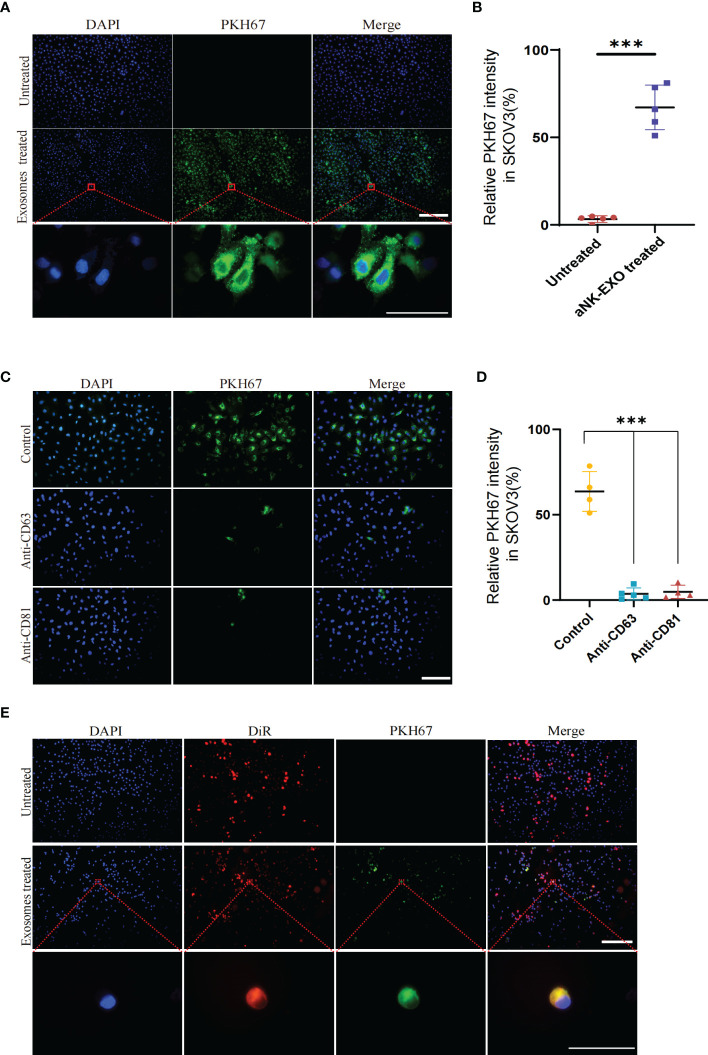
Cellular uptake results of eNK-EXO by OC cells. **(A)** eNK-EXO uptake by SKOV3 cells. SKOV3 cells were stained blue by DAPI and eNK-EXO were stained green by PKH67. **(B)** eNK-EXO uptake quantified by green fluorescence intensity (10×, Scale bar=200 µm; 100×, Scale bar=40 µm, n=5, mean ± SEM, ***p< 0.001). **(C)** eNK-EXO uptake by SKOV3 in the presence of anti-CD63 or anti-CD81. SKOV3 cells were stained blue by DAPI and eNK-EXO were stained green by PKH67 (20×, scale bar=100 µm). **(D)** eNK-EXO uptake quantified by green fluorescence intensity (n=4, Mean ± SEM, t-test, ***p< 0.001). **(E)** Uptake of PKH67-labeled eNK-EXO in the co-culture system of IOSE80 cells and DiR-labeled SKOV3 cells (10×, scale bar=200 µm; 10×, scale bar = 40 µm).

To evaluate whether eNK-EXO can be taken up by OC cells, PKH67-labeled eNK-EXO were incubated with SKOV3 cells for 6 hours and fluorescence images were taken to measure cellular uptake of eNK-EXO (**Figure 3A**). Quantification of fluorescence intensity showed significant uptake of eNK-EXO by SKOV3 cells and the uptake rate could reach 60% in 6 hours (**Figure 3B**). To investigate which receptors are responsible for the uptake, we pretreated SKOV3 cells with anti-CD63 or anti-CD81 antibodies and discovered that either blocking CD63 or CD81 could cause a significant decrease in the proportion of cells that took up PKH67-labeled eNK-EXO (**Figures 3C, D**). These results indicated that CD63 and CD81 might play an important role in the uptake of eNK-EXO by SKOV3 cells, however, other mechanisms may also be involved.

Based on the result that eNK-EXO were cytotoxic to OC cells but had no cytotoxic effect on normal cells (**Figure 2C**), we hypothesized that it could be attributed to the different uptake rates between tumor cells and normal cells. To test our hypothesis, DiR-labeled SKOV3 cells and IOSE80 cells were co-cultured at a ratio of 1:1 and treated with PKH67-labeled eNK-EXO for 6 hours. As shown in **Figure 3E**, preferential uptake of eNK-EXO by SKOV3 cells over IOSE80 was clearly seen as green fluorescence of eNK-EXO readily appeared in DiR-labeled SKOV3 cells while little was seen in IOSE80 (**Figure 3E**).

### The anti-tumor effect of eNK-EXO loaded DDP *in vitro*

**Figure 4:**
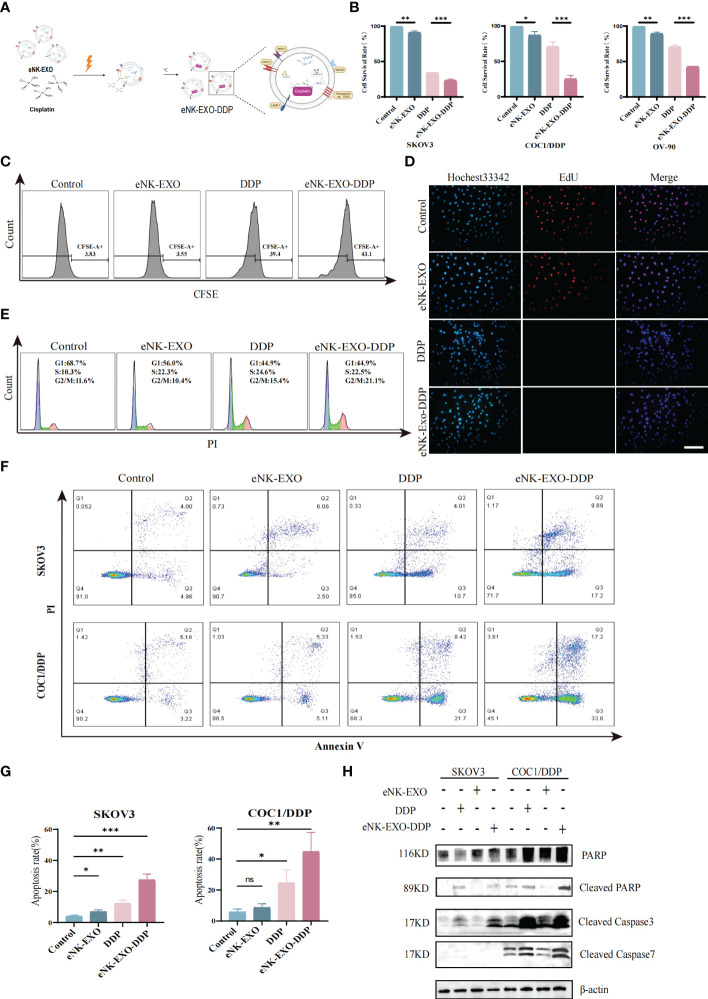
Anti-tumor activity of eNK-EXO-DDP *in vitro*. **(A)** Schematic diagram showing the preparation of eNK-EXO-DDP. **(B)** CCK-8 assay showing the cytotoxicity of eNK-EXO-DDP against SKOV3, COC1/DDP and OV-90 cells (n=3, mean ± SEM, **p< 0.01, ***p< 0.001). **(C)** Flow cytometry analysis of anti-proliferation activity of eNK-EXO-DDP against SKOV3 cells. **(D)** EdU assay result of anti-proliferation activity of eNK-EXO-DDP against SKOV3 cells, Scale bar=100 µm. **(E)** Cell cycle analysis of SKOV3 cells after eNK-EXO-DDP treatment by flow cytometry. **(F)** Apoptosis analysis of SKOV3 cells after eNK-EXO-DDP treatment by flow cytometry. **(G)** Statistical analysis of apoptosis rate is based on three experiments, mean ± SEM, *p< 0.05, **p< 0.01, ***p< 0.001. **(H)** Western blot analysis of PARP, cleaved PARP, cleaved caspase 3 and cleaved caspase 7 in SKOV3 and COC1/DDP cells after different treatments.

To date, dozens of studies have been carried out on the use of exosomes as drug-delivery vehicles (35, 36). Motivated by the excellent performance of exosomes in tumor therapeutic drug delivery, we next explored the potential of using eNK-EXO as delivery vehicles for OC chemotherapeutic drug DDP for OC treatment (**Figure 4A**).

We assayed the anti-tumor effect of eNK-EXO-DDP, eNK-EXO and free DDP alone against SKOV3, OV90, and COC1/DDP ovarian cancer cells in parallel. As shown in **Figure 4B**, compared with eNK-EXO and DDP treated alone, the cell survival rate of three OC cell lines decreased significantly after eNK-EXO-DDP treatment in the CCK-8 assay, especially in DDP-insensitive OV-90 cells and DDP-resistant COC1/DDP cells (**Figure 4B**). These results indicate that eNK-EXO could sensitize OC cells to the effect of DDP. Next, we used flow cytometry and EdU proliferation assay to detect the inhibitory effect of eNK-EXO-DDP on the proliferation of OC cells. Flow cytometry showed that eNK-EXO-DDP significantly suppressed the proliferation of SKOV3 cells, the fluorescence intensity of CFSE decreasing with each generation of cell proliferation (**Figure 4C**). However, due to the efflux of fluorescent dyes by tumor cells (37, 38), we can’t detect the double peak of fluorescence intensity, and the inhibition of proliferation was judged by the degree of rightward shift of the single peak. Moreover, EdU staining also confirmed that eNK-EXO-DDP has a potent inhibitory effect on OC cells (**Figure 4D**).

DDP exerts its antitumor activity by binding to genomic DNA or mitochondrial DNA to block the production of DNA and arrest DNA replication which finally led to apoptosis (4, 39–41). We asked if the anti-tumor effect of eNK-EXO-DDP was the result of DDP activity. Cell cycle analysis showed considerable G2/M arrest in eNK-EXO-DDP treated cells (**Figure 4E**). Apoptosis analyzed by flow cytometry also confirmed that eNK-EXO-DDP can induce apoptosis in SKOV3 cell (27.41% ± 3.12% vs12.29% ± 1.84%) (**Figures 4F, G**) and the proportion of apoptotic cells was comprised of both late apoptotic (upper right quadrant) and early apoptotic cells (bottom right quadrant). eNK-EXO-DDP treatment could induce significant apoptosis in COC1/DDP cells as well (44.72% ± 10.13% vs 24.56% ± 6.87%). Western blot confirmed the increased expression of cleaved PARP, cleaved caspase-3, and cleaved caspase-7 in two OC cell lines upon eNK-EXO-DDP treatment (**Figure 4H**). These results suggest that eNK-EXO-DDP inhibits cell proliferation by inducing cell cycle arrest and apoptosis in OC cells, demonstrating the ability of eNK-EXO to deliver chemotherapeutic drugs to OC cells and also sensitize the cells to drug treatment. However, eNK-EXO alone did not show an anti-tumor effect, which may be because the dose of eNK-EXO used in the above experiment is only 10 µg/mL, which is not enough to exert its anti-tumor effect. As shown in **Figures 2B–E**, the anti-tumor effect of eNK-EXO at this concentration is almost negligible.

### eNK-EXO enhances the cytotoxicity of NK cells

**Figure 5:**
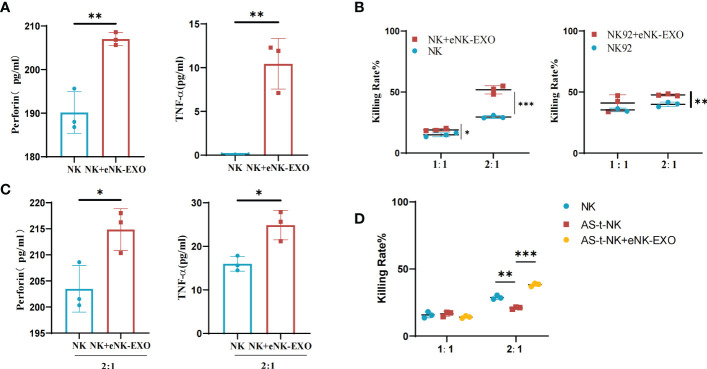
Immunomodulatory effects of eNK-EXO *in vitro*. **(A)** ELISA measurement of perforin and TNF-α concentrations in the growth medium of eNK-EXO treated NK cells (n=3, mean ± SEM, **p< 0.01). **(B)** CCK-8 assay showing the cytotoxicity of eNK-EXO treated NK cells and NK92/MI cells against SKOV3 cells (n=3, mean ± SEM, t-test, *p< 0.05, **p< 0.01, ***p< 0.001). **(C)** ELISA measurement of perforin and TNF-α concentrations in the growth medium of SKOV3 cells co-cultured with eNK-EXO treated NK cells (n=3, mean ± SEM, *p< 0.05). **(D)** CCK-8 assay showing the cytotoxicity of AS-t-NK cells and eNK-EXO treated AS-t-NK cells against SKOV3 cells (n=3, mean ± SEM, **p< 0.01, ***p< 0.001).

Based on the immunomodulatory activity of exosomes, we investigated the effect of eNK-EXO on NK cells. ELISA results showed that NK cells treated with eNK-EXO released more perforin and TNF-α than control (**Figure 5A**). Cytotoxicity of NK cells and NK92/MI cells pre-treated with eNK-EXO was also significantly improved when co-cultured with SKOV3 cells at a 2:1 E:T ratio as measured by CCK-8 (**Figure 5B**). Increased TNF and perforin release by NK cells were also detected by ELISA when NK cells were pre-treated with eNK-EXO and co-cultured with SKOV3 at a 2:1 E:T ratio (**Figure 5C**).

To mimic NK cells in OC TME, we treated NK cells with 10% ascites collected from 7 different ovarian cancer patients. The obtained AS-t-NK cells were assayed for their cytotoxicity against SKOV3 by CCK-8. As shown in **Figure 5D**, the killing effect of AS-t-NK cells on SKOV3 cells was significantly inhibited at a 2:1 E:T ratio. However, treating with eNK-EXO reversed the inhibitory effect and resulted in an even higher cytotoxicity of AS-t-NK cells than that of eNK cells (**Figure 5D**). Therefore, current data suggest that eNK-EXO may be able to re-activate the cytotoxic function of NK cells once impaired in TME.

### RNA sequencing reveals that NK cells treated with eNK-EXO or isolated from OC ascites have a vastly different transcriptional landscape compared to NK cells

**Figure 6:**
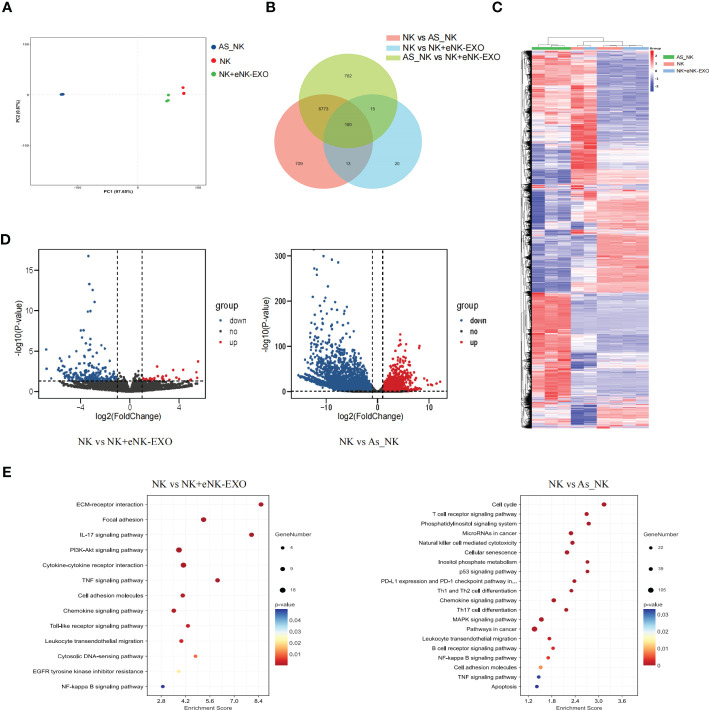
Global transcriptional analysis of AS_NK and eNK-EXO treated NK cells. **(A)** Principal component analysis of eNK, AS_NK, and eNK-EXO treated NK cells. **(B)** Pairwise comparison of all groups. **(C)** Clustering heatmap of all the DEGs in three groups (color scale represents relative gene transcription level). **(D)** Volcano plots showing the distribution of significance [-log_10_(*p*-value)] and the fold changes in the transcription levels of DEGs [log2(fold change)] within different groups. **(E)** Bubble chart showing the functional analysis of DEGs when different groups were compared.

Global transcriptional analysis by RNA-seq was performed to understand the molecular mechanisms underlying the functional difference between NK cells, AS_NK cells and eNK-EXO treated NK cells. Triplicated samples for three groups were subjected to RNA-seq analysis. PCA showed that 3 NK cell samples, 3 AS_NK cell samples, and 3 eNK-EXO treated NK cell samples clustered together (**Figure 6A**), suggesting that most of the observed transcriptional changes are related to the conditions under which the NK cells were treated, with relatively low variability within groups. Then, we performed a pairwise comparison of each group to identify DEGs between groups (**Figure 6B**). A heatmap was generated for samples and genes derived *via* hierarchical clustering (**Figure 6C**). Hierarchical clustering across all samples showed that the samples can be classified into two main groups, NK cells vs the other two groups. By comparing AS_NK cells to NK cells, upregulation of 4933 genes and downregulation of 2742 genes were observed while by comparing eNK-EXO treated NK cells to NK cells, upregulation of 196 and downregulation of 32 genes were observed. An adjusted *p* value threshold of<0.05 was assigned by DESeq2 algorithm (**Figure 6D**). KEGG pathway enrichment analysis revealed that the most enriched DEGs sets in AS_NK vs NK were genes involved in natural killer cell mediated cytotoxicity, and the most enriched DEGs sets in eNK-EXO treated NK vs NK cells are genes involved in cytokine-cytokine interaction, IL-17 signaling pathway, chemokine signaling pathway and TNF signaling pathway (**Figure 6E**).

**Figure 7:**
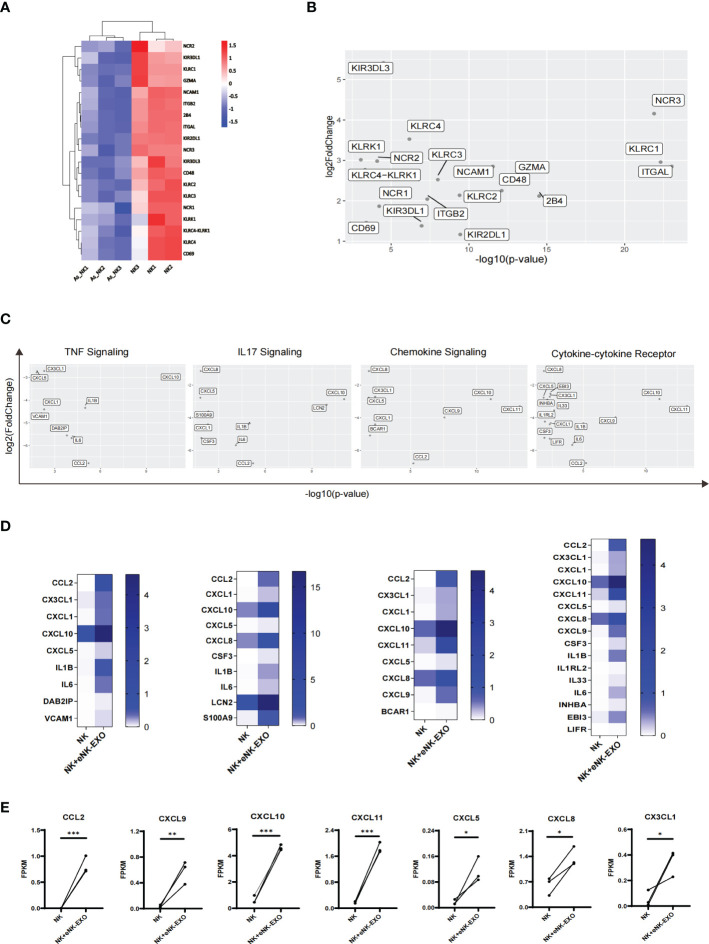
Transcriptional alterations in genes related to pathways that control NK cell function. **(A)** Heatmap of transcription levels of identified DEGs associated with NK cell cytotoxicity. Each column represents an individual in the indicated group. **(B)** Scatter plot displaying genes related to NK cell cytotoxicity. Axis values represent the distribution of significance and changes of expression in AS_NK cells compared to NK cells. **(C)** Scatter plot displaying genes related to signaling pathways. Axis values represent the distribution of significance and changes of expression in eNK-EXO treated NK cells compared to NK cells. **(D)** Heatmaps displaying FKPM values of genes graphed in **(C)**. **(E)** Line graphs showing the transcriptional changes in genes related to activation, migration or tumor killing activity of NK cells. The paired t-test was used to determine significance (* *p*< 0.05, ** *p*< 0.01, *** *p*< 0.001).

A detailed look at DEGs related to the natural killer cell-mediated cytotoxicity pathway in KEGG database revealed considerably lower expression of the following genes *GZMA, CD244 (2B4), CD48, NCR1 (NKP46), NCR2 (NKP44), NCR3 (NKP30), KLRC1 (NKG2A), KLRC2 (NKG2C), KLRC3 (NKG2E), KLRC (NKG2F), KIR2DL1, KIR3DL1* and *KIR3DL3* in AS_NK cells compared to the NK cells (**Figure 7A**). Among them, *NCR3, KLRC1* and *ITGAL* were most significantly down-regulated (**Figure 7B**), which may explain the altered cytotoxicity and functional defects of AS_NK cells.

For eNK-EXO treated NK cells, which exhibited increased cytotoxicity than control NK cells, RNA-seq analysis showed four most significantly up-regulated pathways, the upregulated genes from which were shown in the scatter plot (**Figure 7C**). In addition, heatmaps were generated based on each gene’s expression (**Figure 7D**). Compared with NK cells, most significantly up-regulated genes in eNK-EXO treated NK cells include CXC motif chemokine ligand 10 (*CXCL10*), *CXCL11* and C-C Motif Chemokine Ligand 2 (*CCL2*) (**Figures 7D, E**). Remarkably, the data revealed a substantial increase in the transcription levels of chemokine ligands including *CXCL5*, *CXCL8*, *CXCL9* and *CX3CL1* (**Figure 7E**). Chemokine ligands play a major role in the four up-regulated pathways. For example, CXCR3 is expressed on activated NK cells and functions to enhance NK cell cytotoxicity and promote NK cell proliferation and homing to tumor sites, through binding to its ligands (22, 42–45). CXCR3 ligands, CXCL9, CXCL10 and CXCL11 were up-regulated in NK cells treated with eNK-EXO (**Supplementary Figure S2**). Taken together, eNK-EXO treatment resulted in altered expressions of multiple chemokine ligands on NK cells, which may be one of the mechanisms that lead to phenotypic changes and enhanced cytotoxicity.

## Discussion

Immunotherapies have gained increasing attention in cancer research over the past few decades. NK cell transplantation in the treatment of a variety of solid and haematological tumors is being widely evaluated in clinical trials (reference). However, adoptive therapy with NK cells is often limited by storage conditions, cell transportation and TME inhibition from solid tumors (46). Compared with NK cells, NK-Exo are more convenient to store and transport. Exosomes are usually stored at 20°C for the short term and -80°C for long term storage. The freeze-drying method is also used to preserve exosomes, facilitate its development as a therapeutic drug. Besides, NK-Exo may retain anti-tumor activity because of their lacking the signalling and metabolic pathways that respond to inhibitory TME (21). However, special care should be taken to ensure the quality and quantity of anti-tumor contents in NK-Exo which are unstable and related to the source and state of original NK cells.

In this study, we demonstrate that eNK-EXO have typical NK cell markers and contents, and can kill SKOV3, OV90 and COC1/DDP ovarian cancer cells in a dose-dependent manner. Interestingly, eNK-EXO at the same concentration have no cytotoxic effect on normal cells, indicating its safety profile as cancer therapy. A recent study showed that an antibody blocking the exosome surface marker CD63 could suppress the cytotoxicity of EXO301 (exosomes isolated from oncolytic adenovirus infected HCT116 cells) (47). Therefore, we used anti-CD63 and anti-CD81 antibodies in the cellular uptake experiment, and found that either blocking CD63 or CD81 could significantly decrease the uptake of eNK-EXO by SKOV3 cells. This suggests that the death of ovarian cancer cells is initiated by the internalization of eNK-EXO. Moreover, since eNK-EXO killed OC cells but not normal cells in this study, we hypothesized that OC cells and normal cells have different uptake efficiency of eNK-EXO and confirmed it by the co-culture experiment. However, the detailed mechanism of the differential uptake of eNK-EXO is still unclear. Exosomes could be internalized by directly interacting with extracellular receptors or by direct fusion with the plasma membrane, or a combination of both (36, 48, 49). Further studies are required to understand which mode of internalization contributes to the observed difference in eNK-EXO uptake between SKOV3 and IOSE80 cells.

Exosomes have natural stability, low immunogenicity and excellent tissue/cell penetration (22), and is expected to become an advanced platform for drug/gene delivery (12). Owing to their unique properties, exosomes are currently under evaluation as vehicles for the transport of tumor therapeutic drugs (12). For example, intravenous administration of MSCs is often retained in the lung or liver, while MSC-exo can avoid this problem while still maintaining the therapeutic function of their originating cells (35, 36). 16 clinical studies are investigating the application of MSC-exo in various diseases (50). For tumor treatment, MSC-exo containing siRNA targeting oncogenic *Kras^G12D^* mutations are being investigated in clinical trials to treat pancreatic cancer (NCT03608631) (51, 52). Based on the advantages of using exosomes as drug delivery carrier and the anti-tumor activity of eNK-EXO (53, 54), we evaluated eNK-EXO as a carrier for tumor therapeutic drugs. eNK-EXO-DDP effectively killed OC cells including DDP-insensitive OV90 cells and DDP-resistant COC1/DDP cells, suggesting that eNK-EXO could sensitize tumor cells to DDP. The resistance of tumor cells to DDP may be related to reduced drug uptake, reduced drug inflow or increased drug efflux, drug target changes, and DNA repair (41, 55). In our study, we demonstrated that eNK-EXO-DDP induced cell cycle arrest and apoptosis in OC cells. Therefore, we speculate that the enhanced killing effect of eNK-EXO-DDP on OC cells, especially on COCI/DDP cells is caused by increased uptake of eNK-EXO-DDP by OC cells.

Exosomes have emerged as mediators to reshape the cellular programs of recipient cells. Previous studies have shown exosomes derived from dendritic cells (DCs) and NK cells can exert immunomodulatory effect on target cells. DCs-derived exosomes not only express functional transmembrane MHC and co-stimulatory molecules that enable them to indirectly stimulate adaptive T-cell response, but also express functional transmembrane TNF superfamily ligands (TNFSFLs), including TNF, FasL, and TRAIL, enabling them to directly activate NK cells (56). NK-Exo also contain a variety of proteins involved in immune regulation and can induce the expression of HLA-DR and costimulatory molecules on the surface of monocytes, up-regulate the expression of CD25 and down-regulate the expression of PD-1 on T cells (10), and increase the percentage of CD56^+^ total NK cells and CD56^bright^ and CD56^dim^ subpopulations of NK cells (10, 57). In addition, the above immunomodulatory effects were not affected in the mimicked immunosuppressive environment (10). In our study, eNK-EXO treated eNK cells and NK92 cells showed enhanced tumor-killing activity and increased release of TNF-α and perforin. Interestingly, eNK-EXO treated AS-t-NK cells regained their cytotoxicity against OC cells, which was even stronger than that of NK cells without ascites treatment. The possible molecular mechanism underlying this phenomenon might be explained by the DEGs in the natural killer cell mediated cytotoxicity pathway revealed by RNA-seq analysis, however, how the differential expression of these genes contributes to their varied cytotoxicity required further investigations.

In addition, RNA-seq analysis of eNK-EXO treated NK cells shows significant upregulation of many genes for cytokines that are involved in the regulation of NK cells proliferation, cytotoxicity, and migration, including *CXCL8, CXCL9, CXCL10, CXCL11, CCL2, CXCL5*, and *CX3CL1*. Among them, *CXCL9, CXCL10*, and *CXCL11*, which are significantly up-regulated, are ligands of chemokine receptor CXCR3 (42, 58). CXCR3 is expressed on activated NK cells and participates in physiological processes, such as enhancing NK cells cytotoxicity, promoting NK cell proliferation, and homing to tumor sites, through binding with its corresponding ligands (44, 45). Therefore, we hypothesized that eNK-EXO treated NK cells could enhance their anti-tumor activity and increase the infiltration of NK cells in TME, and change “cold tumor” to “hot tumor” to enhance the effect of tumor immunotherapy. Although the eNK-EXO treated AS_NK cells were not included in the RNA-seq analysis, from the data we have, eNK-EXO can enhance the anti-tumor activity of AS-t-NK cells *in vitro* and up-regulate the expression of genes related to the function of NK cells. Therefore, eNK-EXO could potentially contribute to reversing immune suppression by rescuing defective NK cells in TME.

**Figure 8:**
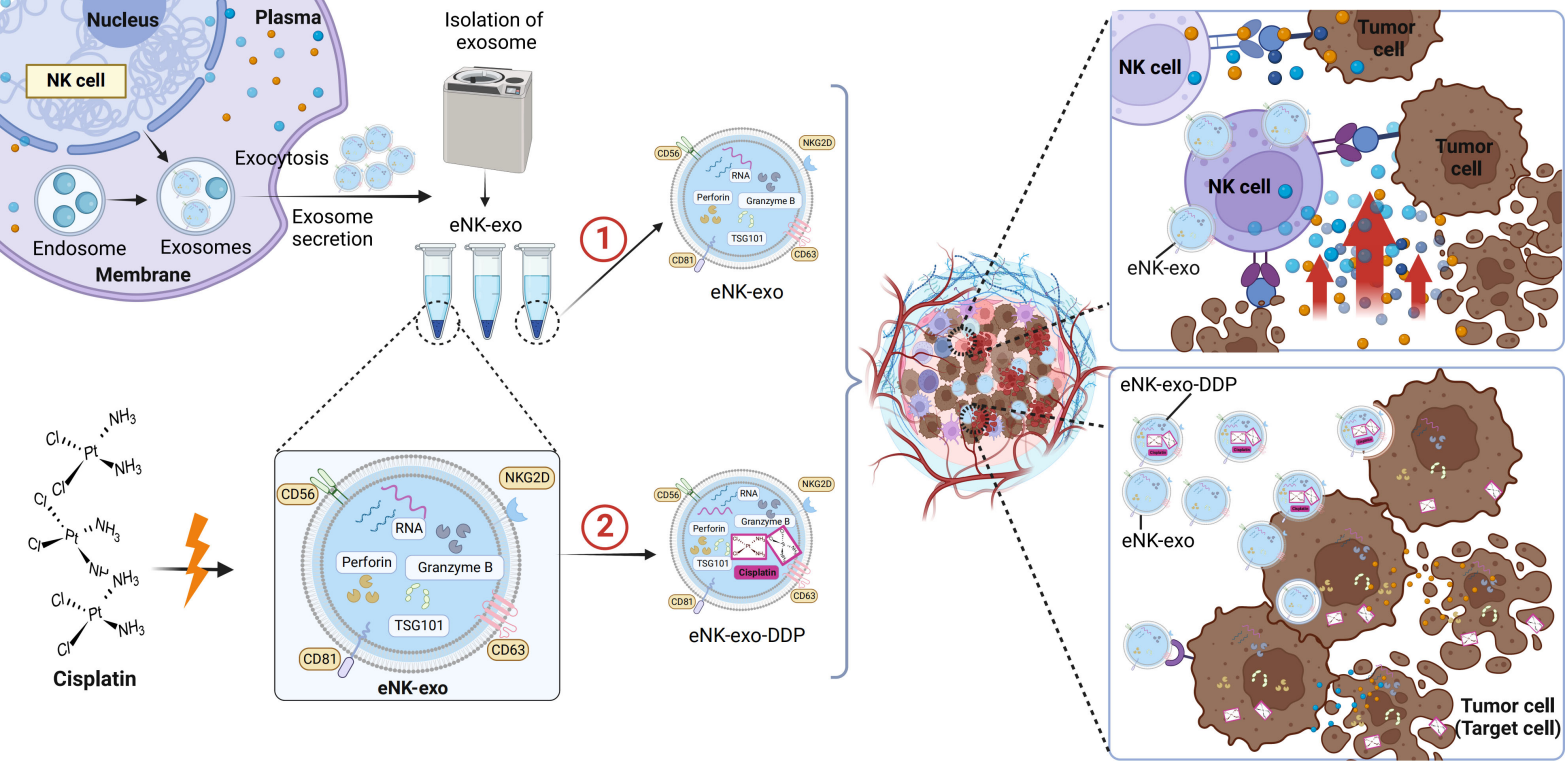
Schematic illustration of eNK-EXO enhance the anti-tumor effects against ovarian cancer by delivering chemotherapeutic drugs and reactivating NK cell functions. ① eNK-EXO containing typical NK cell markers and cytotoxic substances derived from eNK cells show cytotoxicity against OC cells *in vitro*. ② eNK-EXO loaded with cisplatin could sensitize OC cells to the effect of cisplatin and enhance NK cell’s anti-tumor activity.

Taken together, we demonstrated the potential application of eNK-EXO in anti-tumor therapy of OC *in vitro* (**Figure 8**). Being cytotoxic by itself, eNK-EXO can not only deliver and enhance the killing effect of DDP on OC cells but also reverse the immunosuppression of NK cells, thus enhancing its anti-tumor activity. To our knowledge, this is the first report on eNK-EXO-based drug delivery and improvement of immunosuppressive capacity for OC therapy. However, our study were limited to the cellular level due to large amount of exosomes required for *in vivo* study. Much work is still required to clarify the detailed mechanisms by which eNK-EXO enhances the cytotoxicity of NK cells. Nonetheless, our work may represent a starting point for further evaluation of eNK-EXO in solid tumor therapies.

## Data availability statement

The datasets presented in this study can be found in online repositories. The names of the repository/repositories and accession number(s) can be found in the article/**Supplementary Material**.

## Ethics statement

The studies involving human participants were reviewed and approved by the Ethical Committee of GuiZhou Medical University (NO.2022-82). The patients/participants provided their written informed consent to participate in this study.

## Author contributions

XZ and ZZ designed the study,wrote and critically reviewed the manuscript. HL and JD performed the experiments and wrote the manuscript. AY, YHZ and SL was responsible for the data collection and statistical analysis. XL expanded and characterized NK cells. ZY and QC isolated AS_NK cells from ascites. NY and YY prepared the figures. TG, DZ, YCZ, JZ, WO and WY provided the CB of donors, ascites of patients and patient informed consent. All authors contributed to the article and approved the submitted version.
