# Supplementary material for: NK cell-derived exosomes enhance the anti-tumor effects against ovarian cancer by delivering cisplatin and reactivating NK cell functions
Source: Front Immunol. 2023 Jan 19;13:1087689. doi: 10.3389/fimmu.2022.1087689 (PMC9892755; doi:10.3389/fimmu.2022.1087689)
Supplement: Supplementary file 1 [file DataSheet_1.pdf]

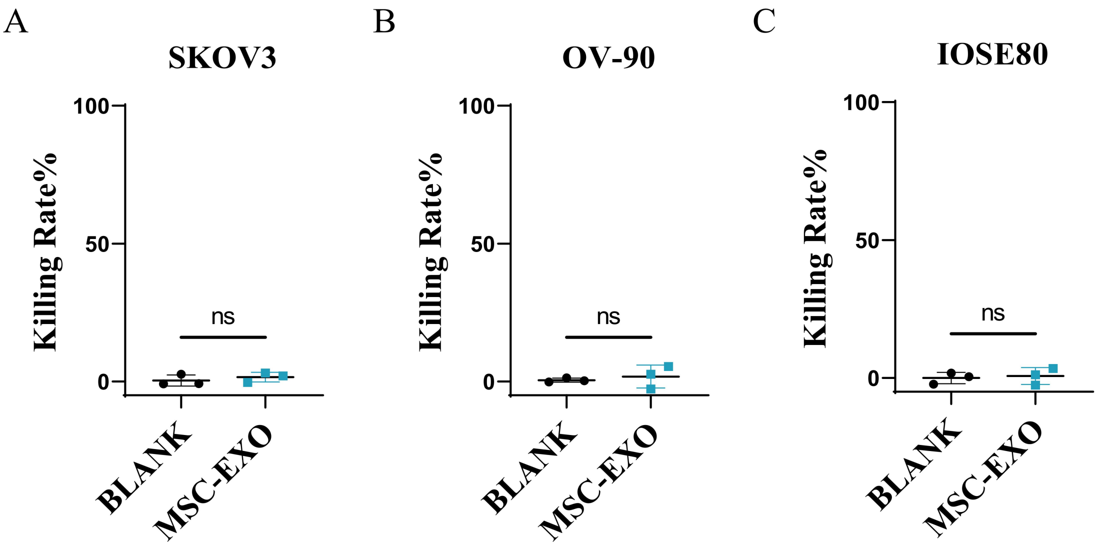

Supplementary Figure S1 Anti-tumor activity of MSC-exo *in vitro*. CCK-8 assay showing the cytotoxicity of MSC-exo against SKOV3**(A)**, OV-90**(B)** and IOSE80 **(C)** (n=3, mean ± SEM).

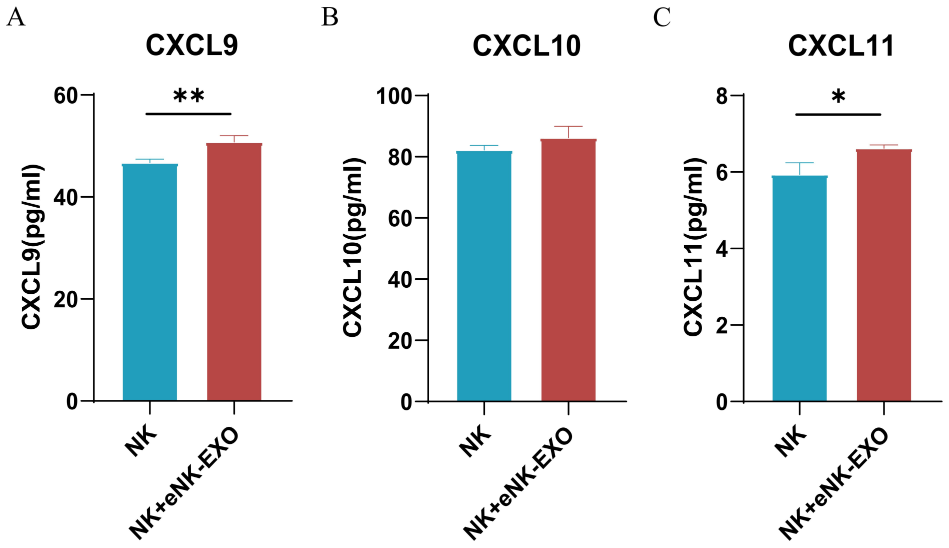

Supplementary Figure S2 ELISA measurement of CXCL9**(A)**, CXCL10**(B)** and CXCL11**(C)** level in the supernatants of eNK-EXO treated NK cells (n=3, mean ± SEM, **$p < 0.05$,**$p < 0.01$).

Supplementary Table S1 The clinicopathological features of 7 ovarian cancer patients.

NO.	Age（year）	Pathologic type	FIGO stage	Peritoneal metastasis
#1	33	Malignant ovarian cancer	IV	Yes
#2	53	High-grade serous carcinoma	IIIC	Yes
#3	45	Mucinous cystadenocarcinoma	IC	NO
#4	63	Clear cell carcinoma	IIA	Yes
#5	55	High-grade serous carcinoma	IIIC	Yes
#6	69	High-grade serous carcinoma	IIIC	Yes
#7	44	High-grade serous carcinoma	IIIC	Yes
