# Supplementary material for: NK cell-derived exosomes enhance the anti-tumor effects against ovarian cancer by delivering cisplatin and reactivating NK cell functions
Source: Front Immunol. 2023 Jan 19;13:1087689. doi: 10.3389/fimmu.2022.1087689 (PMC9892755; doi:10.3389/fimmu.2022.1087689)
Supplement: Supplementary file 2 [file DataSheet_2.docx]

The datasets presented in this study can be found in online repositories.

All the experimental raw data mentioned in the article and the process of re-analyzing RNAseq data according to the analysis requirements were uploaded to Figshare database. The raw data were sorted according to different picture sequences, and were also separately classified according to different experiments. Here are the link addresses of all the data:

luo, heyong (2022): ELISA. figshare. Dataset. https://doi.org/10.6084/m9.figshare.21441681.v1

luo, heyong (2022): EdU assay. figshare. Figure. https://doi.org/10.6084/m9.figshare.21441663.v1

luo, heyong (2022): Flow cytometry analysis. figshare. Dataset. https://doi.org/10.6084/m9.figshare.21441510.v2

luo, heyong (2022): CCK8. figshare. Dataset. https://doi.org/10.6084/m9.figshare.21441564.v1

luo, heyong (2022): RNAseq re-analysis. figshare. Dataset. https://doi.org/10.6084/m9.figshare.21456465.v1

luo, heyong (2022): Western blot. figshare. Journal contribution. https://doi.org/10.6084/m9.figshare.21441432.v1

luo, heyong (2022): Fig8. figshare. Dataset. https://doi.org/10.6084/m9.figshare.21440415.v1

luo, heyong (2022): Fig7. figshare. Dataset. https://doi.org/10.6084/m9.figshare.21440166.v1

luo, heyong (2022): Fig6. figshare. Dataset. https://doi.org/10.6084/m9.figshare.21439245.v1

luo, heyong (2022): Fig5D. figshare. Dataset. https://doi.org/10.6084/m9.figshare.21438993.v1

luo, heyong (2022): Fig5C. figshare. Dataset. https://doi.org/10.6084/m9.figshare.21438759.v1

luo, heyong (2022): Fig5B. figshare. Dataset. https://doi.org/10.6084/m9.figshare.21438597.v1

luo, heyong (2022): Fig5A. figshare. Dataset. https://doi.org/10.6084/m9.figshare.21438492.v1

luo, heyong (2022): Fig4G(PARP). figshare. Figure. https://doi.org/10.6084/m9.figshare.21438300.v1

luo, heyong (2022): Fig4G(cleaved PARP). figshare. Figure. https://doi.org/10.6084/m9.figshare.21438282.v1

luo, heyong (2022): Fig4G(cleaved caspase7). figshare. Figure. https://doi.org/10.6084/m9.figshare.21438273.v1

luo, heyong (2022): Fig4G(cleaved caspase3). figshare. Figure. https://doi.org/10.6084/m9.figshare.21438267.v1

luo, heyong (2022): Fig4G(beta-actin). figshare. Figure. https://doi.org/10.6084/m9.figshare.21438252.v1

luo, heyong (2022): Fig4F. figshare. Dataset. https://doi.org/10.6084/m9.figshare.21438222.v1

luo, heyong (2022): Fig4E. figshare. Dataset. https://doi.org/10.6084/m9.figshare.21438219.v1

luo, heyong (2022): Fig4D. figshare. Figure. https://doi.org/10.6084/m9.figshare.21438159.v1

luo, heyong (2022): Fig4C. figshare. Dataset. https://doi.org/10.6084/m9.figshare.21438150.v1

luo, heyong (2022): Fig4B. figshare. Dataset. https://doi.org/10.6084/m9.figshare.21438141.v1

luo, heyong (2022): Fig4A. figshare. Figure. https://doi.org/10.6084/m9.figshare.21438120.v1

luo, heyong (2022): Fig3E. figshare. Figure. https://doi.org/10.6084/m9.figshare.21438096.v1

luo, heyong (2022): Fig3D. figshare. Dataset. https://doi.org/10.6084/m9.figshare.21438087.v1

luo, heyong (2022): Fig3C. figshare. Figure. https://doi.org/10.6084/m9.figshare.21438066.v1

luo, heyong (2022): Fig3B. figshare. Dataset. https://doi.org/10.6084/m9.figshare.21438060.v1

luo, heyong (2022): Fig3A. figshare. Figure. https://doi.org/10.6084/m9.figshare.21438009.v1

luo, heyong (2022): Fig2E. figshare. Dataset. https://doi.org/10.6084/m9.figshare.21438000.v1

luo, heyong (2022): Fig2D. figshare. Figure. https://doi.org/10.6084/m9.figshare.21437973.v1

luo, heyong (2022): Fig2C. figshare. Dataset. https://doi.org/10.6084/m9.figshare.21437967.v1

luo, heyong (2022): Fig2B(GZMB). figshare. Figure. https://doi.org/10.6084/m9.figshare.21437955.v1

luo, heyong (2022): Fig2B(perforin). figshare. Figure. https://doi.org/10.6084/m9.figshare.21437895.v1

luo, heyong (2022): Fig2B(CDD56). figshare. Figure. https://doi.org/10.6084/m9.figshare.21437730.v1

luo, heyong (2022): Fig2A. figshare. Figure. https://doi.org/10.6084/m9.figshare.21437721.v1

luo, heyong (2022): Fig1D. figshare. Figure. https://doi.org/10.6084/m9.figshare.21437712.v1

luo, heyong (2022): Fig1D. figshare. Figure. https://doi.org/10.6084/m9.figshare.21437697.v1

luo, heyong (2022): Fig1D. figshare. Figure. https://doi.org/10.6084/m9.figshare.21437514.v1

luo, heyong (2022): Fig1D. figshare. Figure. https://doi.org/10.6084/m9.figshare.21437331.v1

luo, heyong (2022): Fig1C. figshare. Figure. https://doi.org/10.6084/m9.figshare.21437214.v1

luo, heyong (2022): Fig1B. figshare. Dataset. https://doi.org/10.6084/m9.figshare.21432186.v1

luo, heyong (2022): Fig1A. figshare. Dataset. https://doi.org/10.6084/m9.figshare.21432177.v1

RNAseq raw data is uploaded to the GEO database, and the GEO accession number is GSE217191.
